# Japanese multicenter database of healthy controls for [^123^I]FP-CIT SPECT

**DOI:** 10.1007/s00259-018-3976-5

**Published:** 2018-02-24

**Authors:** Hiroshi Matsuda, Miho Murata, Yohei Mukai, Kazuya Sako, Hidetoshi Ono, Hiroshi Toyama, Yoshitaka Inui, Yasuyuki Taki, Hideo Shimomura, Hiroshi Nagayama, Amane Tateno, Kenjiro Ono, Hidetomo Murakami, Atsushi Kono, Shigeki Hirano, Satoshi Kuwabara, Norihide Maikusa, Masayo Ogawa, Etsuko Imabayashi, Noriko Sato, Harumasa Takano, Jun Hatazawa, Ryosuke Takahashi

**Affiliations:** 1 Integrative Brain Imaging Center, National Center of Neurology and Psychiatry, 4-1-1 Ogawa-Higashi, Kodaira, Tokyo 187-8551 Japan; 2 Department of Neurology, National Center Hospital of Neurology and Psychiatry, Kodaira, Tokyo Japan; 3 Department of Neurology, Nakamura Memorial Hospital, Sapporo, Japan; 4 Department of Radiology, Nakamura Memorial Hospital, Sapporo, Japan; 5 Department of Radiology, Fujita Health University, Toyoake, Japan; 6 Department of Nuclear Medicine and Radiology, Institute of Development, Aging and Cancer, Tohoku University, Sendai, Japan; 7 Department of Neurology, Graduate School of Medicine, Nippon Medical School, Bunkyo-ku, Tokyo, Japan; 8 Department of Neuropsychiatry, Graduate School of Medicine, Nippon Medical School, Bunkyo-ku, Tokyo, Japan; 9 Department of Neurology, Showa University, Shinagawa-ku, Tokyo, Japan; 10 Department of Radiology, Kobe University, Kobe, Japan; 11 Department of Radiology, National Cerebral and Cardiovascular Center, Suita, Japan; 12 Department of Neurology, Chiba University, Chiba, Japan; 13 Department of Radiology, National Center Hospital of Neurology and Psychiatry, Kodaira, Tokyo Japan; 14 Department of Nuclear Medicine, Osaka University, Osaka, Japan; 15 Department of Neurology, Kyoto University, Kyoto, Japan

**Keywords:** Multicenter trial, SPECT, [^123^I]FP-CIT, Normal database, Dopamine transporter

## Abstract

**Purpose:**

The aim of this multicenter trial was to generate a [^123^I]FP-CIT SPECT database of healthy controls from the common SPECT systems available in Japan.

**Methods:**

This study included 510 sets of SPECT data from 256 healthy controls (116 men and 140 women; age range, 30–83 years) acquired from eight different centers. Images were reconstructed without attenuation or scatter correction (NOACNOSC), with only attenuation correction using the Chang method (ChangACNOSC) or X-ray CT (CTACNOSC), and with both scatter and attenuation correction using the Chang method (ChangACSC) or X-ray CT (CTACSC). These SPECT images were analyzed using the Southampton method. The outcome measure was the specific binding ratio (SBR) in the striatum. These striatal SBRs were calibrated from prior experiments using a striatal phantom.

**Results:**

The original SBRs gradually decreased in the order of ChangACSC, CTACSC, ChangACNOSC, CTACNOSC, and NOACNOSC. The SBRs for NOACNOSC were 46% lower than those for ChangACSC. In contrast, the calibrated SBRs were almost equal under no scatter correction (NOSC) conditions. A significant effect of age was found, with an SBR decline rate of 6.3% per decade. In the 30–39 age group, SBRs were 12.2% higher in women than in men, but this increase declined with age and was absent in the 70–79 age group.

**Conclusions:**

This study provided a large-scale quantitative database of [^123^I]FP-CIT SPECT scans from different scanners in healthy controls across a wide age range and with balanced sex representation. The phantom calibration effectively harmonizes SPECT data from different SPECT systems under NOSC conditions. The data collected in this study may serve as a reference database.

**Electronic supplementary material:**

The online version of this article (10.1007/s00259-018-3976-5) contains supplementary material, which is available to authorized users.

## Introduction

Single-photon emission computed tomography (SPECT) imaging of the dopamine transporter (DAT), which is intensively expressed in the striatum, is widely used in clinical practice to support the diagnosis of dopaminergic degenerative movement disorders such as Parkinson’s disease [1] and the differentiation of dementia with Lewy bodies and Alzheimer’s disease [2]. ^123^I–*N*-ω-fluoropropyl-2β-carboxymethoxy-3β-(4-iodophenyl)nortropane ([^123^I]FP-CIT) is a widely used radiopharmaceutical that binds to the DAT. SPECT imaging using [^123^I]FP-CIT is routinely assessed via visual interpretation by an expert reader in clinical studies. However, in difficult-to-interpret cases, quantification of striatal DAT binding helps clinicians to reach a more confident interpretation of the scan [3] or helps to increase confidence in less-experienced readers [4]. Whether a SPECT scan is considered normal or abnormal sometimes relies on a comparison of individual values to a reference database obtained in healthy controls. This reference database should be constructed from a large number of normal subjects covering a large age range to account for subtle effects such as age-related differences. This large-scale normal database is best achieved by using a multicenter framework; however, multicenter trials using SPECT are affected by many issues. Differences among scanner types/models and collimators, together with differences among scanning protocols, reconstruction algorithms, and quantitative analysis methods, mean that cross-center comparisons of quantitative outcome measures of DAT density would not be possible without some sort of harmonization [5–8].

The present project was undertaken to generate a large-scale database of [^123^I]FP-CIT SPECT scans of healthy controls from the most common SPECT systems available in Japan using a multicenter approach. An additional aim was to generate cross-calibration factors to harmonize quantitative outcome measures of DAT density obtained with different SPECT systems.

## Materials and methods

### Phantom study

In eight participating centers, phantom studies were applied to 17 imaging devices: four GE Infinia systems, two GE 670, one GE 640, three Toshiba 9300R, two Toshiba ECAM, two Siemens Symbia T6, one Siemens Symbia T16, one Siemens Symbia, and one Philips Bright View (Table 1). These imaging devices were multiple detector systems (triple- or dual-head) combined with or without X-ray computed tomography (CT). Collimators included low-energy high-resolution, fan-beam, and ones specifically designed for ^123^I brain SPECT, low- to medium-energy general-purpose or extended low-energy general-purpose. The acquisition parameters were as follows: matrix 128 × 128; angular sampling ≤6° (360°); and hardware zoom (1.0–1.45) to achieve a pixel size of 1.7–3.4 mm. Besides the photopeak imaging window (159 KeV ± 10%, 143–175 KeV), two additional scatter energy windows were also acquired below and above the main window to assess the value of scatter correction.

**Table 1:** Details of the healthy controls recruited in each center and the SPECT system used for imaging

Center	Men (*n*)	Women (*n*)	Age (years)		Combinations of SPECT scanner + Collimators	
			Mean ± SD	Range	Combination of first scan (no. of subjects)	Combination of second scan (no. of subjects)
Chiba	9	21	64 ± 23	41–82	GE Infinia + ELEGP (18)	GE Infinia + LEHR (18)
Toyoake	14	22	70 ± 6	55–82	Toshiba 9300R + fan-beam (36)	Siemens Symbia T6 or T16 + LMEGP (36)
Kobe	25	3	38 ± 7	30–63	GE Optima NM/CT 640 + ELEGP (19)	Toshiba ECAM + LMEGP (18)
Sapporo	20	20	61 ± 12	38–83	Toshiba 9300R + fan-beam (40)	Toshiba ECAM + LMEGP (40)
Tokyo (Kodaira)	19	21	55 ± 27	30–81	Siemens Symbia T6 + LMEGP (23)	GE Discovery NM/CT 670 pro+ ELEGP (23)
Tokyo (Shinagawa)	10	16	52 ± 27	35–83	Toshiba 9300R + fan-beam (26)	GE Discovery NM/CT 670 + LEHR (26)
Tokyo (Bunkyo)	14	16	57 ± 14	31–79	GE Infinia + LEHR (28)	Philips BrightView + Cardiac High Resolution (27)
Sendai	5	21	58 ± 12	30–72	Siemens Symbia + LMEGP (13)	GE Infinia + LEHR (13)
Total	116	140	57 ± 15	30–83		

*LEHR* low-energy high-resolution, *LMEGP* low- to medium-energy general-purpose, *ELEGP* extended low-energy general-purpose, *SD* standard deviation

The numbers refer to the subjects and scans included in the analysis of the SPECT data

Pool and line source phantoms filled with ^123^I solution were used to assess the SPECT uniformity and the spatial resolution of the different imaging systems, respectively. In addition, ^123^I SPECT images of an anthropomorphic striatal phantom (NMP Business Support Co., Ltd., Hyogo, Japan) were acquired in the same manner as in a previous report [9]. The striatal phantom was filled with different ^123^I concentrations between the striatal compartments and the background to reflect the possible binding observed in clinical investigations with the radiopharmaceutical [^123^I]FP-CIT (Nihon Medi-Physics Co., Ltd., Tokyo, Japan). Accordingly, the nominal ratios ranged from highly normal to very low (abnormal) and null (uniform filling) radiotracer binding, namely, 8:1, 6:1, 4:1, and 3:1.

The striatal phantom was initially filled uniformly, using a solution with a concentration of ~5 kBq/mL. For the next filling, which aimed to achieve striatal to background ratios of 8:1 and 4:1 in the right and left striatum, respectively, the background compartment was left untouched while the striatal compartments were emptied and refilled using solutions with concentrations of ~40 kBq/mL for the right striatum and ~20 kBq/mL for the left. By increasing the background concentration by 33% and leaving the striatal compartments untouched, additional ratios of 6:1 and 3:1 were achieved. Aliquots of 1 mL were taken from the background and striatal compartments in each experiment and measured in the well counter available at each center.

### Subjects

This study was registered in the University Hospital Medical Information Network Clinical Trials Registry (UMIN000016811). In total, 291 subjects (132 men and 159 women; age range, 30–83 years) were recruited from eight participating centers. All participants were Japanese and were healthy according to medical history, neurological examination, including the Unified Parkinson’ Disease Rating Scale (UPDRS) [10], and psychiatric evaluation, including the Beck Depression Inventory ver. I (BDI-I) [11] or ver. II (BDI-II) [12] scales. General cognition was assessed by means of the Japanese version of the Montreal Cognitive Assessment (MOCA-J) [13]. Handedness was assessed with the Edinburgh Inventory [14].

Inclusion criteria were (1) a UPDRS part III score of 0 in subjects younger than 60 years or <5 in older subjects; (2) a score < 10 on the BDI-I or score < 12 on the BDI-II; (3) a score ≥ 26 on the MOCA-J; and (3) fully normal MRI in subjects younger than 60 years; in older subjects, white matter hyperintensities in T2-weighted images or fluid-attenuated inversion recovery, corresponding to a white matter lesion score < 3 on the Fazekas scale [15], were accepted, provided that the basal ganglia was spared.

Exclusion criteria were (1) an abnormality in the neurological examination; (2) dementia diagnosed by the Diagnostic and Statistical Manual of Mental Disorders (DSM)-5 [16]; (3) history of Parkinsonism in first-degree relatives (siblings, parents, or children); (4) contraindication for MRI; (5) use of medicine that influences the accumulation of ^123^I–ioflupan in the brain (cocaine, mazindol, methylphenidate, selective serotonin reuptake inhibitors) [17, 18]; (6) a rapid eye movement sleep behavior disorder (RBD) evaluated by a single question session for RBD and the Japanese version of the RBD screening questionnaire [19]; (7) allergy to alcohol or iodide; and (8) pregnancy, lactation, or possibility of becoming pregnant.

Twenty-six subjects were excluded during the screening due to the inclusion and exclusion criteria in terms of MOCA-J, BDI, UPDRS part III, RBD, allergy to alcohol, or contraindication for MRI (cosmetic tattoo). Two of the subjects who passed the screening withdrew their consent before the MRI examination because of intolerance to MRI examination and physical deconditioning. The three-dimensional whole-brain T1-weighted, T2-weighted, and FLAIR images were excluded from one subject because of an occult cerebral infarct. After the MRI examination, six subjects withdrew informed consent because of pregnancy, physical deconditioning, or change of mind. Finally, 256 subjects (116 men and 140 women; age range, 30–83 years) underwent the SPECT studies (Table 1). Of these, 243 were right-handed (110 men and 133 women), 12 were left-handed (6 men and 6 women), and one was ambidextrous (1 woman).

### SPECT imaging

[^123^I]FP-CIT (168 ± 5 MBq, 158–186 MBq, specific activity ranged from 5.85 × 10^11^ to 10.5 × 10^11^ Bq/mg) was injected intravenously. Two serial SPECT scans were started at 3.1 ± 0.2 and 3.8 ± 1.1 h after tracer injection using different SPECT scanners in an individual subject. The protocol for the acquisition of projection data and reconstruction for each SPECT system was the same as in the phantom experiment (see Supplementary material). One of two SPECT data was not available in two male subjects due to technical difficulties. Accordingly, 510 SPECT data were obtained.

The total scan time ranged from 27 to 42 min. The median time was 28 min. Projection data from fan-beam collimators were rebinned to parallel projection data. Projection data were filtered by Butterworth filtering with a cutoff of 0.55–0.76 cm^−1^ and order of 4 to 10. Reconstruction of projection data was performed using filtered back projection or an ordered-subset expectation-maximization (OSEM) algorithm.

Five kinds of reconstructions were performed: with neither attenuation nor scatter correction (NOACNOSC), with only attenuation correction using Chang’s method under an attenuation coefficient of 0.07–0.11 cm^−1^ (ChangACNOSC) or X-ray CT (CTACNOSC), and with both scatter correction and attenuation correction using Chang’s method under an attenuation coefficient of 0.07–0.146 cm^−1^ (ChangACSC) or X-ray CT (CTACSC).

The reconstructed data were quantified using the Southampton method [20], which involves collecting the whole radioactivity from the striatum of each hemisphere and estimating the background radioactivity from the whole brain minus that from the striatum. This analysis is reported to be less dependent of partial volume effects, which would improve the consistency in quantitative measurements between centers with different imaging devices. The specific-to-nondisplaceable binding ratio (SBR) was defined as:

1 $$ \mathrm{SBR}=\frac{\mathrm{Cs}}{Cr} $$

where Cs is the count concentration in the striatum due to the specific binding only and Cr is the count concentration in a reference region due to the non-specific binding. In the Southampton method, SBR is calculated from sufficiently large volume of interest (VOI) including all counts associated with striatal activity to be independent from the size of the VOI and from the resolution of the SPECT system as follows:

2 $$ \mathrm{SBR}=\frac{1}{{\mathrm{C}}_{\mathrm{per}\ \mathrm{pixel}\ \mathrm{BG}\ \mathrm{VOI}}}\left(\frac{{\mathrm{C}}_{\mathrm{Str}\mathrm{VOI}}-{\mathrm{C}}_{\mathrm{per}\ \mathrm{pixel}\ \mathrm{BG}\ \mathrm{VOI}}\ \mathrm{X}\ \mathrm{Number}\ \mathrm{of}\ {\mathrm{pixel}}_{\mathrm{Str}\mathrm{VOI}}}{{\mathrm{Vol}}_{\mathrm{Str}}}\right) $$

where C_StrVOI_ is the count of the striatum VOI, C_perpiexl BG VOI_ is the count of the reference VOI per pixel, number of pixel_StrVOI_ is number of pixels in the striatum VOI, and Vol_Str_ is the volume of the striatum (fixed to 11.2 ml). Reference VOI was automatically defined over the cerebral cortex on the transaxial slices within a slab 44 mm thick centered on the highest striatal activity. Then, the absolute values of the asymmetry index (AI) were calculated as follows:

3 $$ \mathrm{AI}=\left[\frac{\mathrm{R}-\mathrm{L}}{\mathrm{R}+\mathrm{L}}\right]\ast 200 $$

where R and L represent the right and left striatal SBR, respectively.

Originally measured SBRs in each subject under three or five reconstruction conditions by the Southampton method are calibrated using the relationship between measured SBRs of SPECT data of a phantom and the true SBR from measurements of aliquots.

### Statistical analysis

Differences in the SBR among reconstruction conditions with or without attenuation and scatter correction were evaluated using intraclass correlation coefficients (ICCs) to validate the effects of calibration using the phantom study. Differences in the average SBR and AI for the right and left striatum were evaluated using univariate analysis of variance (UNIANOVA) to validate the effects of age, sex, and start time of SPECT scanning. Moreover, multiple regression analysis was performed to investigate the relationships of the average SBR or the AI of the SBR with age, sex, and start time of SPECT scanning. Statistical analysis was conducted using IBM SPSS Statistics for Windows, Ver. 22 (IBM Corp.). All effects with significance levels less than 0.05 were examined.

## Results

**Fig. 1:**
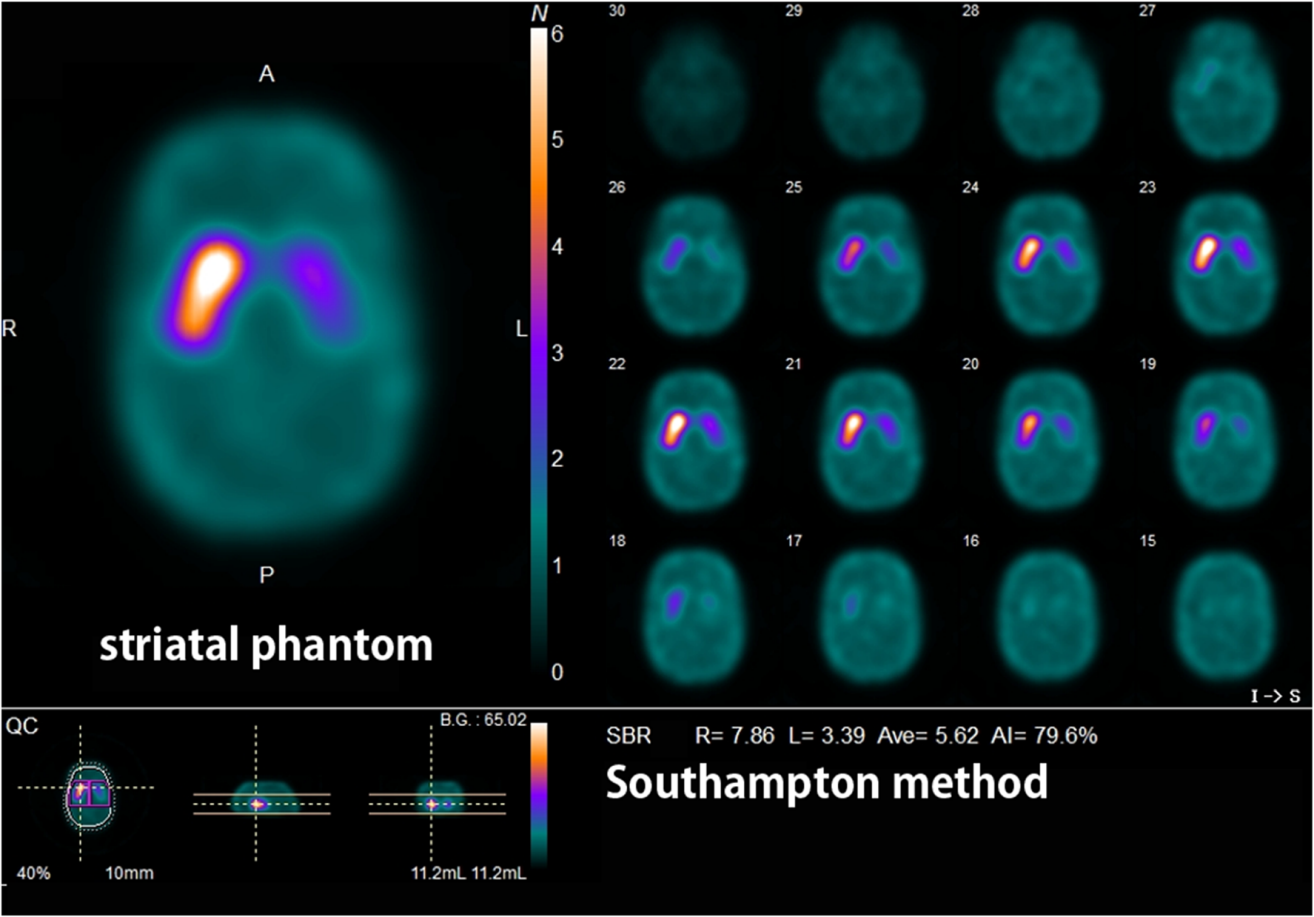
SPECT image of a striatal phantom containing different ^123^I activity concentrations between striatal compartments and the background. Striatal SBRs were estimated by the Southampton method

**Fig. 2:**
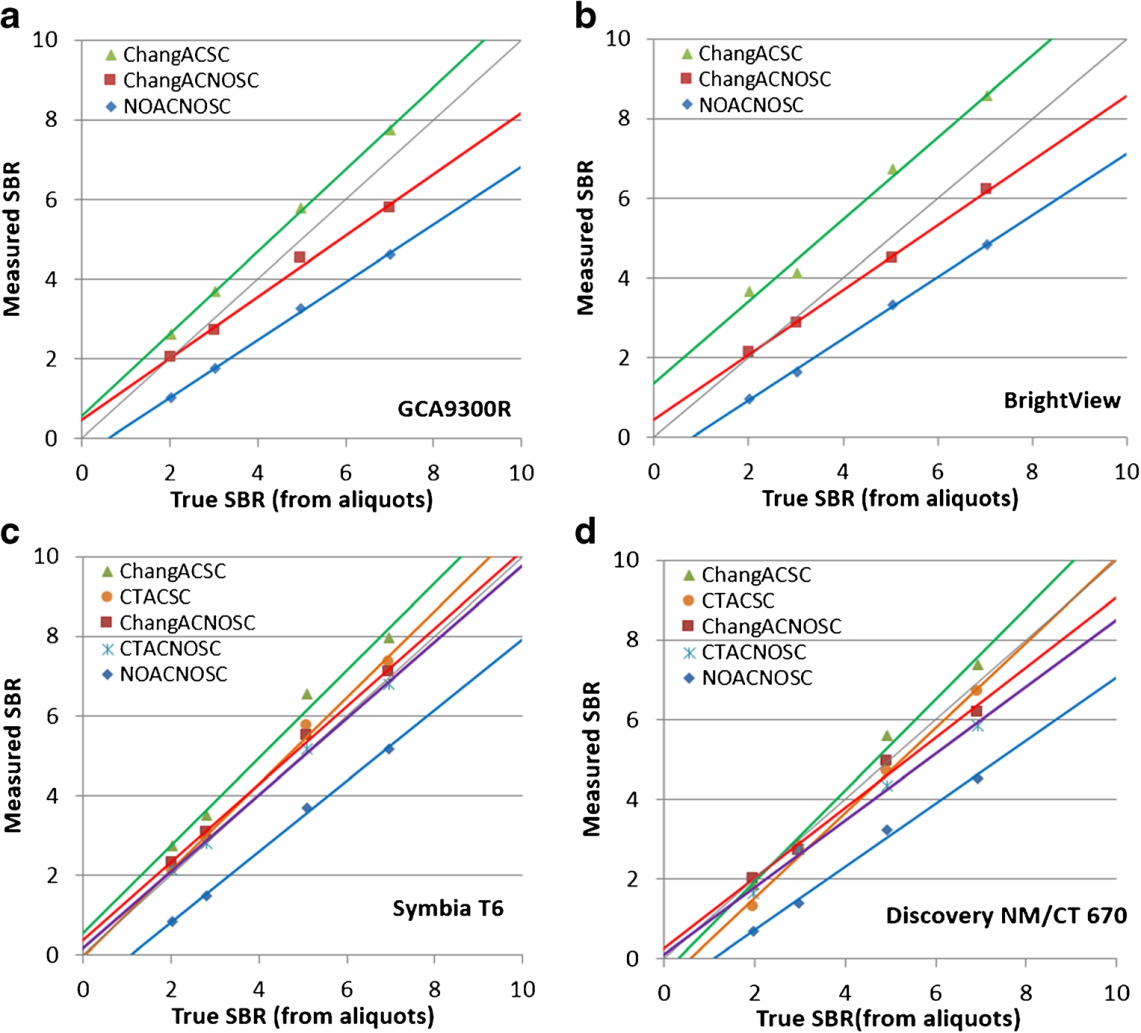
Measured SBRs of SPECT data of a phantom by the Southampton method plotted against the true SBR from measurements of aliquots by a well counter for the Toshiba GCA9300A (**a**), Philips Bright View (**b**), Siemens Symbia T6 (**c**), and GE Discovery MN/CT 670 (**d**). The linear regression lines are displayed for each reconstruction condition, namely, ChangACSC, ChangACNOSC, CTACSC, CTACNOSC, and NOACNOSC. Also shown is the line of identity (in *gray*). Similarly high correlations were also obtained between the measured SBR and true SBR in other SPECT scanners

**Fig. 3:**
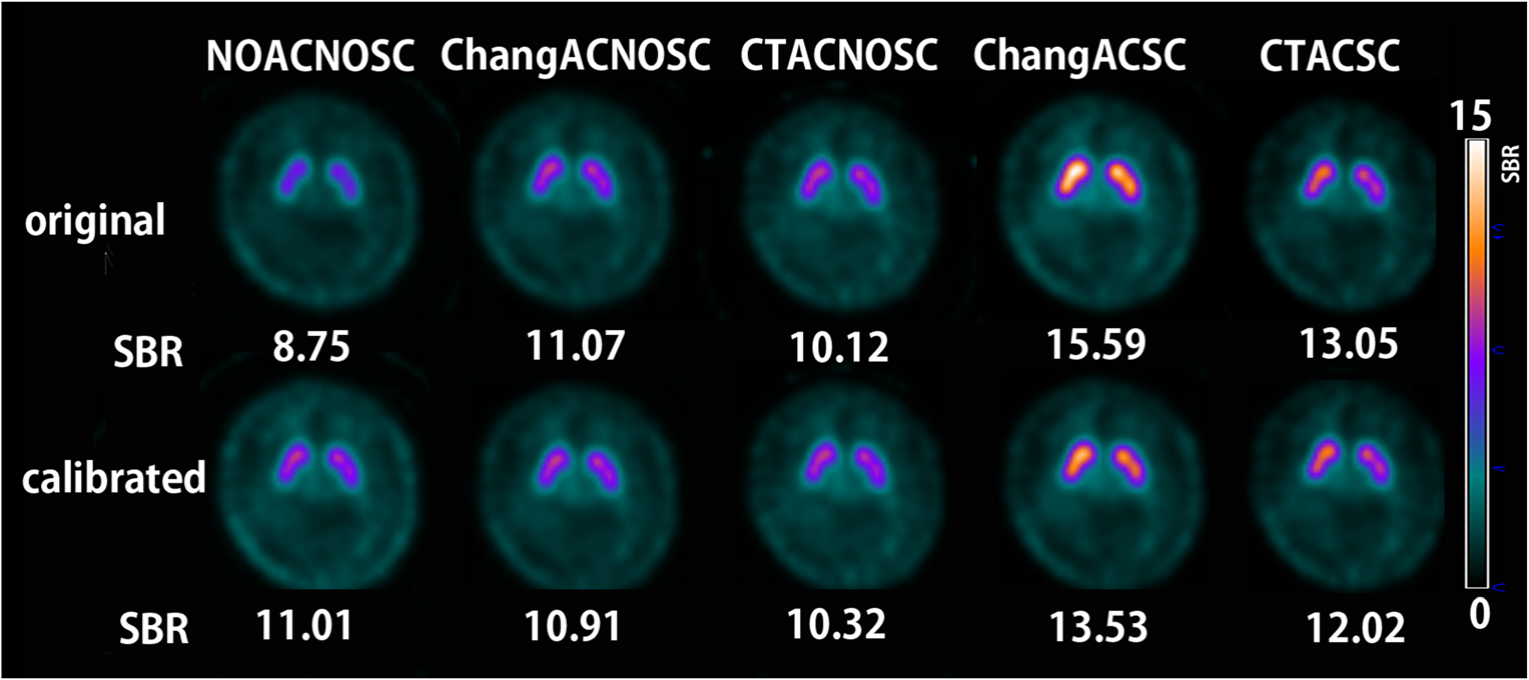
Quantitative SPECT images before and after phantom calibration in a young healthy control under different reconstruction conditions. The original SBRs before calibration ranged widely from 8.75 in NOACNOSC to 15.59 in ChangACSC. In contrast, the calibrated SBRs were almost constant under NOSC conditions and somewhat higher under scatter correction conditions

The true SBR obtained by measurements of aliquots using a well counter and the SBR measured by Southampton analysis of a SPECT image from a striatal phantom (Fig. 1) showed a linear relation in all SPECT scanners (Fig. 2). Linear regression equations for calibration are listed for all SPECT scanners with different reconstruction conditions (see Supplementary material). The original and calibrated SBR values using these linear regression equations measured in healthy controls are listed in Table 2. Original SBR values gradually decreased in the order of the reconstruction conditions of ChangACSC, CTACSC, ChangACNOSC, CTACNOSC, and NOACNOSC. Calibrated SBR values showed lesser gradual decrease than original SBR values in the order of the reconstruction conditions of ChangACSC, CTACSC, NOACNOSC, ChangACNOSC, and CTACNOSC. The original and calibrated SBR values for NOACNOSC were 46% and 12% lower than those for ChangACSC, respectively. SBRs under conditions of scatter correction showed somewhat higher values than those obtained under NOSC conditions even after calibration. When SBRs under conditions with attenuation correction and/or scatter correction were compared with those for NOACNOSC, the ICCs were much higher and with much narrower confidence intervals after calibration under NOSC conditions. Representative images of quantitative DAT SPECT before and after calibration are shown in Fig. 3.

**Table 2:** Correlation of original and calibrated SBRs and intraclass correlation coefficients (ICCs) between NOACNOSC (X-axis) and other reconstruction conditions (Y-axis) in healthy controls

Reconstruction conditions	Number of SPECT scans	Average SBR	Range (min, max)	Linear regression equation	ICC (ρ)	95% confidence interval of ICC (ρ)
Original						
CTACSC	292	9.12	4.41, 14.31	Y = 1.200X + 2.334	0.438	−0.096, 0.781
ChangACSC	510	10.48	5.41, 19.08	Y = 1.445X + 2.138	0.333	−0.075, 0.698
CTACNOSC	292	6.72	3.32, 11.46	Y = 0.996X + 1.091	0.860	−0.126, 0.963
ChangACNOSC	510	7.09	3.40, 12.21	Y = 1.121X + 0.618	0.808	−0.159, 0.945
NOACNOSC	510	5.77	2.18, 10.94	NA	NA	NA
Calibrated						
CTACSC	292	8.96	4.37, 13.76	Y = 1.053X + 0.517	0.832	0.372, 0.928
ChangACSC	510	9.31	4.10, 17.05	Y = 1.103X + 0.297	0.820	0.222, 0.928
CTACNOSC	292	7.63	3.35, 11.83	Y = 0.825X + 1.011	0.926	0.839, 0.959
ChangACNOSC	510	7.95	3.97, 14.66	Y = 0.927X + 0.380	0.958	0.940, 0.969
NOACNOSC	510	8.16	3.81, 13.89	NA	NA	NA

*CTACSC* computed tomography attenuation correction with scatter correction, *ChangACSC* Chang attenuation correction with scatter correction, *CTACNOSC* computed tomography attenuation correction without scatter correction, *ChangACNOSC* Chang attenuation correction without scatter correction, *NOACNOSC* without attenuation or scatter correction

**Fig. 4:**
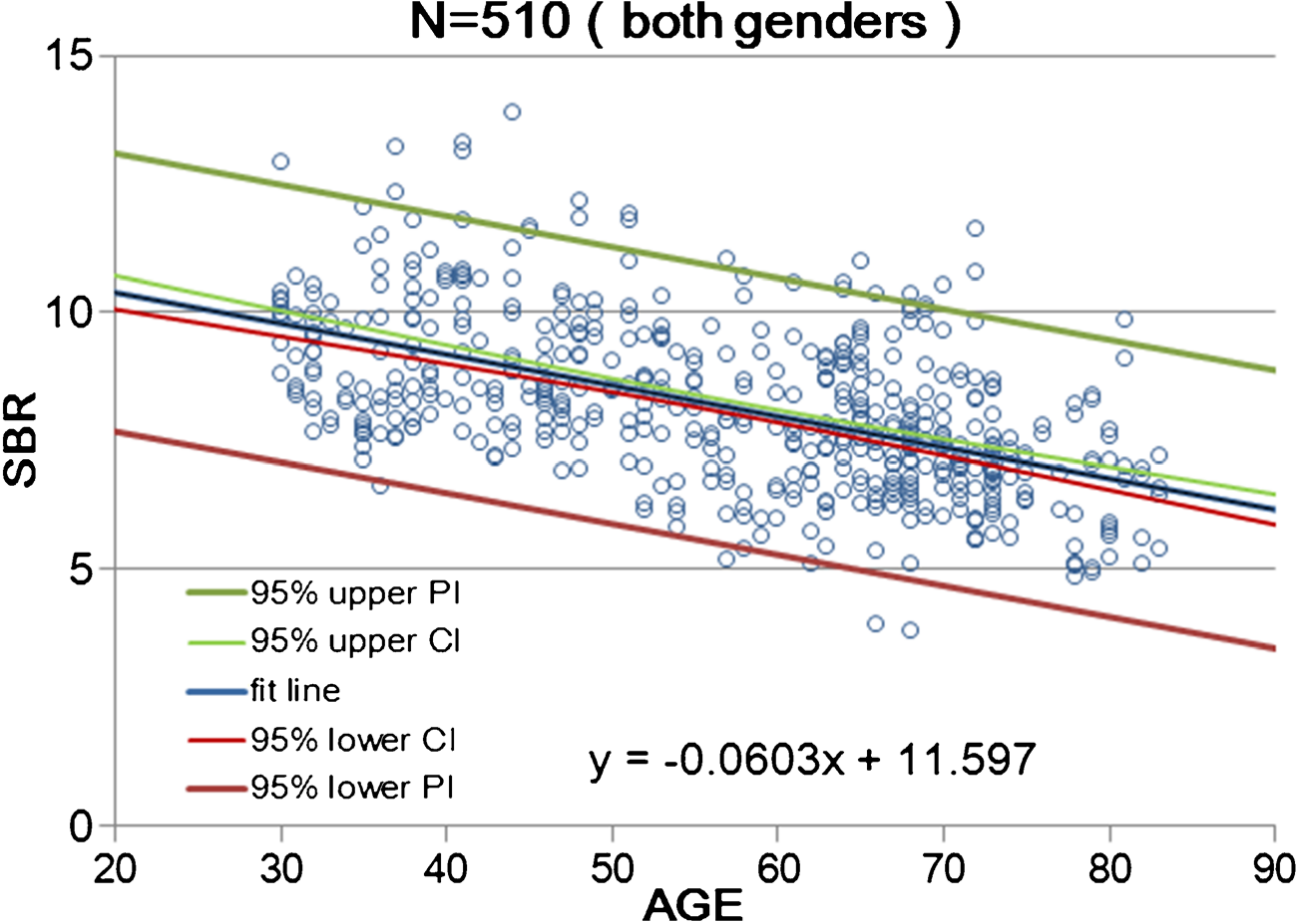
Scatter plot of SBR as a function of age in 510 data from 256 healthy controls of both sexes. Data relevant to the average SBR for the left and right striatum are fitted by a linear regression line with 95% upper and lower confidence interval (CI) and prediction interval (PI) lines

**Fig. 5:**
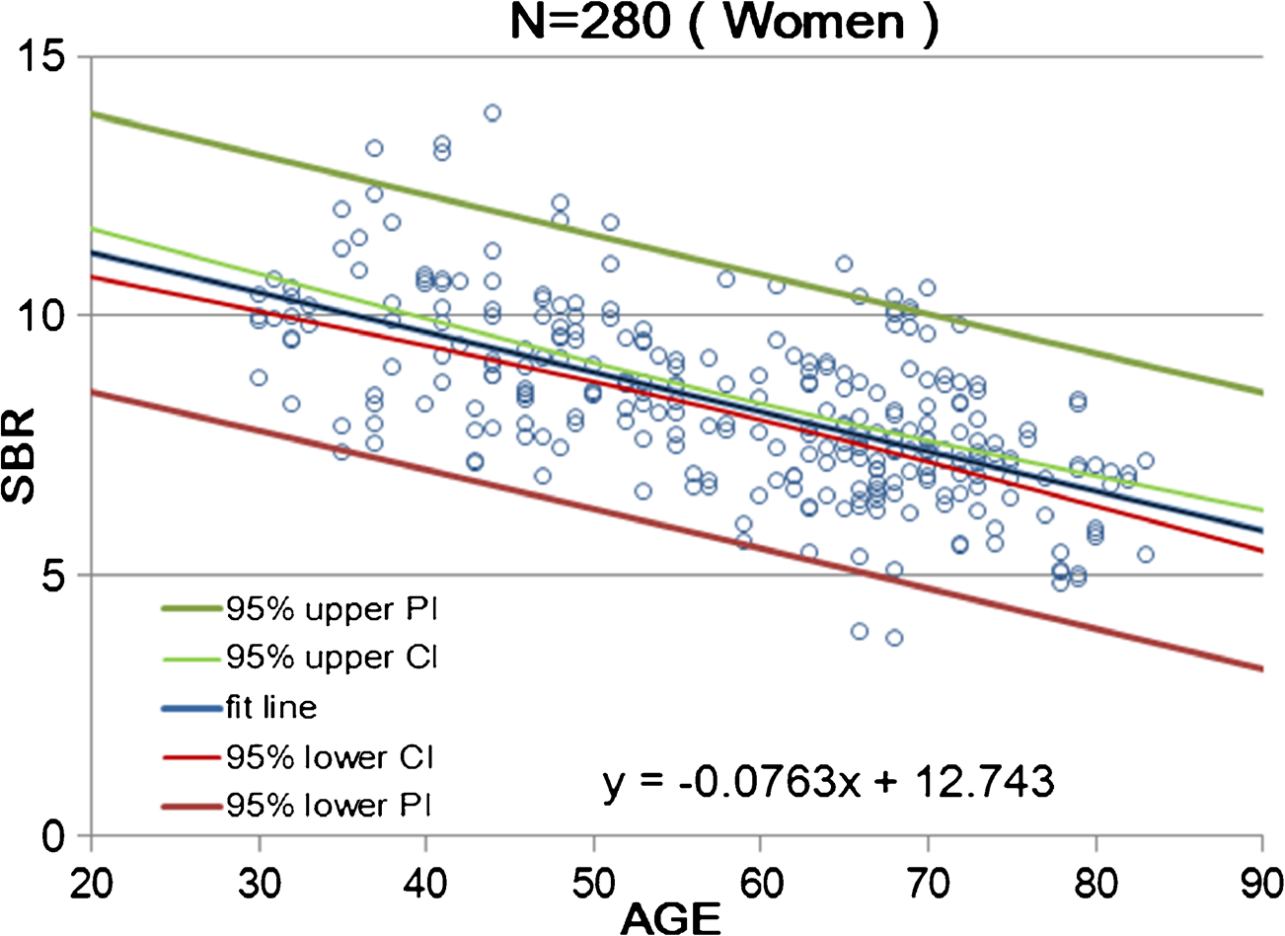
Scatter plot of SBR as a function of age in 280 data from 140 women. Data relevant to the average SBR for the left and right striatum are fitted by a linear regression line with 95% upper and lower confidence interval (CI) and prediction interval (PI) lines

**Fig. 6:**
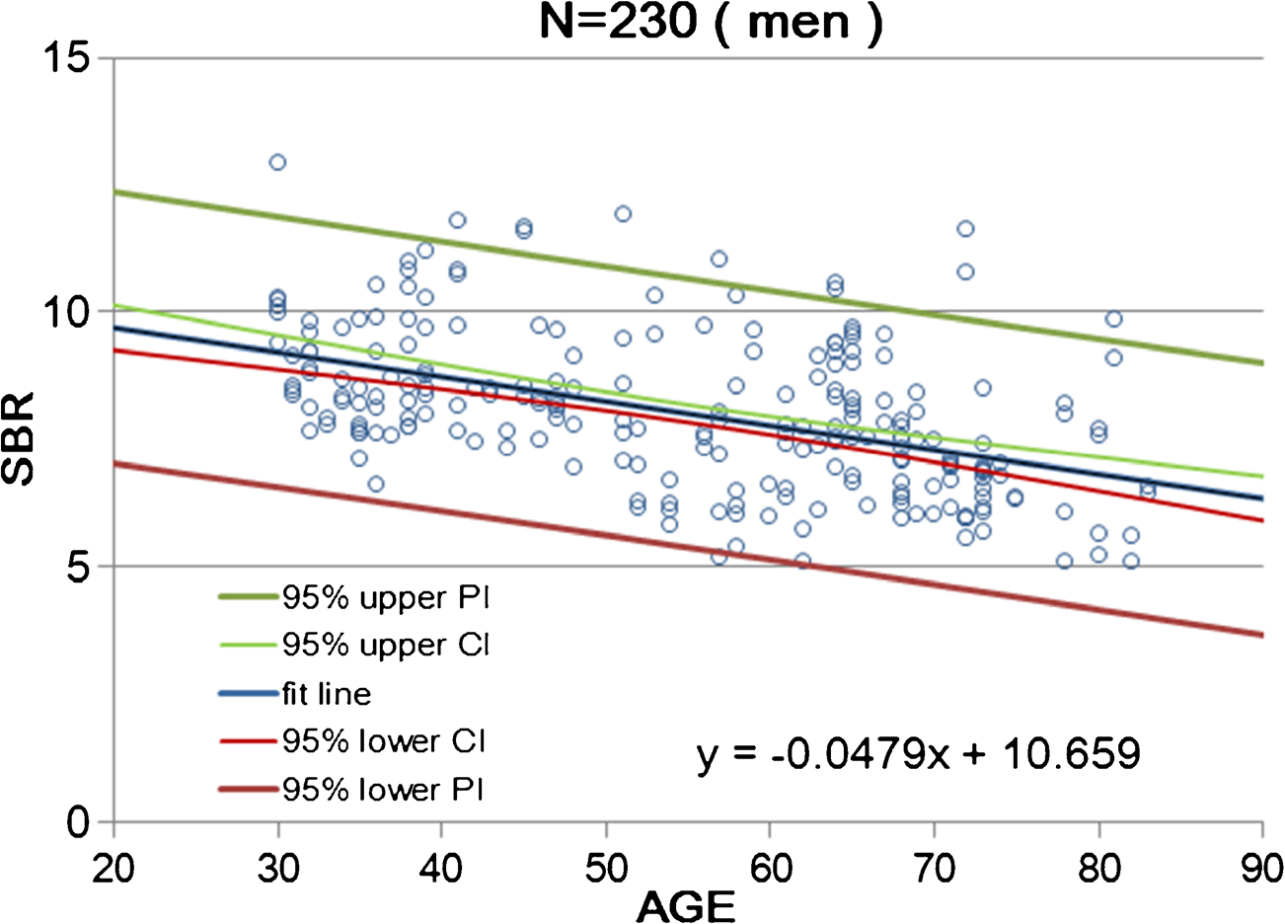
Scatter plot of SBR as a function of age in 230 data from 116 men. Data relevant to the average SBR for the left and right striatum are fitted by a linear regression line with 95% upper and lower confidence interval (CI) and prediction interval (PI) lines

Calibrated SBR values for NOACNOSC revealed a significant linear decline with advancing age (*n* = 510, r = −0.539, *p* < 0.001, Y = −0.0603X + 11.6; Fig. 4). The rate of reduction in SBR per decade was 6.3% on average. Women showed a somewhat steeper decline with advancing age (*n* = 280, r = −0.620, *p* < 0.001, Y = −0.0763X + 12.74; Fig. 5) than men (*n* = 230, r = −0.479, *p* < 0.001, Y = −0.0479X + 10.66; Fig. 6). In the 30–39 age group, women showed 12.2% higher SBR values than men but this increase declined with aging and was not evident in the 70–79 age group (Table 3). The average reduction rate of the SBR for men and women was 5.3% and 7.5% per decade, respectively. UNIANOVA (Table 4) revealed that SBRs were significantly affected by age (F = 234.7, *p* < 0.001), sex (F = 18.6, *p* < 0.001), and start time of SPECT scan after tracer injection (F = 4.07, *p* = 0.044). Moreover, a significant interaction was found between sex and age (F = 11.9, *p* = 0.001). On the basis of multiple regression analysis (Table 4), formulae were determined to enable the estimation of the expected SBR values as follows:

4 $$ \mathrm{SBR}=10.610\hbox{--} 0.063\times \mathrm{age}+0.263\times \mathrm{start}\ \mathrm{time}\ \left(\mathrm{h}\right)+0.461\ \left(\mathrm{if}\ \mathrm{sex}=\mathrm{female}\right) $$

**Table 3:** Calibrated SBRs for NOACNOSC by age group

Parameter	Age group (years)					
	30–39	40–49	50–59	60–69	70–79	80–89
**Both sexes**						
Number of subjects	45	43	39	71	48	10
Number of scans	90	86	78	142	95	19
Average of left and right SBRs	9.52	8.91	8.31	7.71	7.10	6.50
95% lower limit of prediction intervals	6.82	6.22	5.62	5.01	4.41	3.80
Interhemispheric asymmetry index	3.59	3.88	4.17	4.46	4.75	5.04
95% upper limit of prediction intervals	10.53	10.81	11.09	11.38	11.68	11.99
**Men**						
Number of subjects	30	15	17	31	18	5
Number of scans	60	30	34	62	35	9
Average of left and right SBRs	9.01	8.53	8.05	7.57	7.09	6.61
95% lower limit of prediction intervals	6.36	5.89	5.41	4.93	4.44	3.95
Interhemispheric asymmetry index	3.40	3.93	4.46	4.98	5.51	6.04
95% upper limit of prediction intervals	10.58	11.09	11.61	12.14	12.69	13.25
**Women**						
Number of subjects	15	28	22	40	30	5
Number of scans	30	56	44	80	60	10
Average of left and right SBRs	10.11	9.35	8.58	7.82	7.06	6.30
95% lower limit of prediction intervals	7.46	6.71	5.95	5.18	4.42	3.64
Interhemispheric asymmetry index	3.81	3.91	4.00	4.10	4.20	4.30
95% upper limit of prediction intervals	10.55	10.62	10.71	10.81	10.92	11.05

**Table 4:** Univariate analysis of variance and multiple regression analysis of 510 DAT SPECT in healthy volunteers

**Univariate analysis of variance**				
Between-subject effects	Degrees of freedom	Mean square	F	p-value
Sex	1	27.712	2.256	0.134
Age	1	103.041	8.390	0.004
Sex × age	1	49.038	3.993	0.046
Scan start time	1	0.193	0.16	0.900
Error	505	12.281		
R^2^ = 0.028 (R^2^_adj_ = 0.020)				
**Multiple regression analysis**				
Model	B (unstandardized coefficient)	t	p-value	
(Constant)	10.610	22.134	< 0.001	
Age	−0.063	−15.18	< 0.001	
Scan start time	0.263	2.024	0.043	
Sex	0.461	3.819	< 0.001	

The dependent variable is the average SBR for the right and left striatum. Independent variables are sex, age, and scan start time

**Fig. 7:**
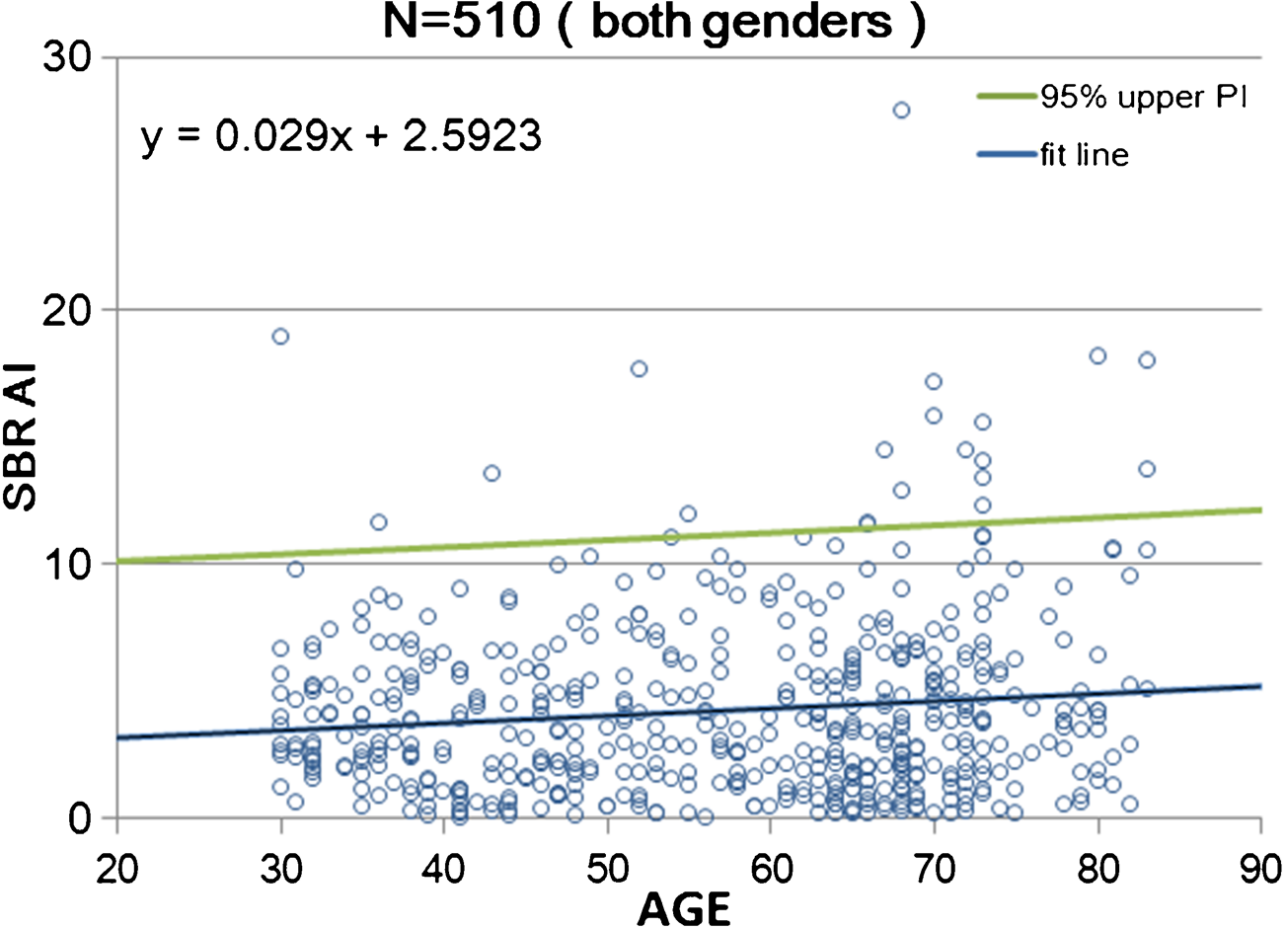
Scatter plot of the asymmetry index (AI) between the right and left striatal SBR as a function of age in 510 data from 256 healthy controls of both sexes. Data relevant to the AI of the striatal SBR are fitted by a linear regression line with a 95% upper prediction interval (PI) line

The AI of the calibrated SBRs for NOACNOSC revealed a significant increase with age (*n* = 510, r = 0.119, *p* < 0.001, Y = 0.029X + 2.592; Fig. 7). In men and women, the right striatal SBR (8.07 and 8.31, respectively, on average) was slightly but significantly (*p* = 0.003 and *p* = 0.0016, respectively) higher than the left striatal SBR (7.97 and 8.24, respectively, on average) in a paired t-test. Men showed a steeper AI increase (*n* = 230, r = 0.216, *p* < 0.001, Y = 0.0528X + 1.58) than women (*n* = 280, r = 0.039, *p* = 0.505, Y = 0.0098X + 3.47) with age. UNIANOVA (Table 5) revealed that the AI of the SBR was significantly affected by age (F = 8.39, *p* = 0.004). Moreover, a significant interaction was found between sex and age (F = 3.99, *p* = 0.046). On the basis of multiple regression analysis (Table 5), the following formula was developed to enable the estimation of the expected SBR values:

5 $$ \mathrm{AI}\ \mathrm{of}\ \mathrm{SBR}=2.885+\hbox{--} 0.031\times \mathrm{age}\hbox{--} 0.546\ \left(\mathrm{if}\ \mathrm{sex}=\mathrm{female}\right) $$

**Table 5:** Univariate analysis of variance and multiple regression analysis of 510 DAT SPECT in healthy volunteers

**Univariate analysis of variance**				
Between-subject effects	Degrees of freedom	Mean square	F	p-value
Sex	1	27.712	2.256	0.134
Age	1	103.041	8.390	0.004
Sex × age	1	49.038	3.993	0.046
Scan start time	1	0.193	0.16	0.900
Error	505	12.281		
R^2^ = 0.028 (R^2^_adj_ = 0.020)				
**Multiple regression analysis**				
Model	B (unstandardized coefficient)	t	p-value	
(Constant)	2.885	2.306	0.022	
Age	0.031	2.875	0.004	
Scan start time	−0.037	−0.108	0.914	
Sex	−0.546	−1.731	0.084	

The dependent variable is the asymmetric index (AI) of the SBR for the right and left striatum. Independent variables are sex, age, and scan start time

## Discussion

This study was designed to generate a large-scale database of [^123^I]FP-CIT SPECT scans of healthy controls across a wide age range and using different SPECT scanners from eight different Japanese centers. The SPECT data were mainly collected using dual-head SPECT systems and triple-head SPECT systems combined with or without X-ray CT, representing the most typical systems currently available in nuclear medicine departments in Japan. To our knowledge, this is the largest database ever reported, comprising 510 scans from 256 subjects for [^123^I]FP-CIT SPECT.

The age-related decline in DAT availability measured in this study was 6.3% per decade on average for both sexes, which is in agreement with previous [^123^I]FP-CIT studies reporting values ranging from 3.6% to 7.5% [21–27] in western countries. Recently, Yamamoto et al. [28] reported a higher reduction of 8.9% per decade in 30 Japanese healthy controls ranging from 50 to 86 years, and they ascribed these higher values to a racial difference in DAT gene polymorphisms. However, the present results obtained from 256 Japanese subjects ranging from 30 to 83 years may show a reduced effect of this racial difference. To describe the influence of age on DAT density, a linear model gave the best fit for our data, which is in agreement with these earlier studies. DAT is expressed in the presynaptic axonal terminals of nigrostriatal pathways. A histopathological investigation [29] reported that neuronal loss linearly occurs at a rate of 4.7–6.0% per decade from the fifth to ninth decades. Another histopathological study also reported a linear drop in pigmented neurons with advancing age in the pars compacta of the caudal substantia nigra at a rate of 4.7% per decade [30]. These almost equivalent rates of reductions between neurons in the substantia nigra and DAT in the striatum suggest that striatal DAT may be associated with nigral dopaminergic neuronal loss with advancing age. The method for SBR measures should also be taken into account. In SBR measures using the Southampton method, striatal volume is fixed to a standard volume of 11.2 ml in all subjects. However, striatal volume has been reported to show annual reduction of approximately 0.8% with advancing age [31]. If we apply the actual striatal volume to Vol_Str_ in Eq. (2), lesser reduction of SBR with advancing age may be obtained. Accordingly, reduction rate of SBR with advancing age may be somewhat overestimated in the present study.

The presymptomatic phase for Parkinson’s disease is taken as the period from the onset of neuronal loss in substantia nigra to the onset of symptoms. It has been reported to be 4.7 years for the total age-adjusted count in Parkinson’s disease [30]. During this period neuronal loss due to Parkinson’s disease, excluding age-related loss, has been estimated to be 31% for the total count. This neuronal loss is almost equivalent to the SBR reduction rate of 35% calculated from the ratio of the 95% lower limit of prediction intervals to the averaged SBR at an age group of 60–69 presented in Table 3.

We observed markedly higher DAT availability in women than in men in younger age groups. Previous studies have already shown such higher DAT availability in women than in men using [^123^I]FP-CIT [21, 23–25, 27, 28]. Premenopausal women have higher striatal presynaptic dopamine synthesis than age-matched men [32]. Estrogen increases presynaptic activity in female rats [33] and the dopamine turnover rate and even the density of dopaminergic innervation are higher in female rats than in male rats [34]. These findings may support the reduction in the greater DAT availability in women with advancing age in the present study. Men have an approximately 7% larger striatal volume than women [35]. If we apply the actual striatal volume to Vol_Str_ in Eq. (2) of the Southampton method, lower and higher SBR values are obtained in men and in women, respectively. Accordingly, the sex difference may be somewhat underestimated in the present study.

Conflicting results have been reported in previous reports in terms of interhemispheric differences in striatal SBRs measured by the Southampton method. Although the present study found right-side dominancy, other investigators have reported left-side dominancy [23] or no interhemispheric differences [21, 27]. However, a previous study [24] reported a similar finding of a mild increase in interhemispheric differences in the measured striatal SBRs with advancing age.

A later scan start time was associated with a slight increase in measured SBR in the present study. In a previous report [24], SPECT data showed a slight but significantly higher SBR when measured at 4 h than at 3 h. This effect might be related to the fact that a perfect transient equilibrium condition had not been achieved at 3 h and thus DAT availability might have been slightly underestimated.

Phantom calibration brought significant changes to the databases, with an increase in the SBRs under NOSC conditions. The aim of the calibration is, in fact, not only to harmonize the differences in performance between different camera models, but also to recover the true SBR values under NOSC conditions. In contrast, phantom calibration decreased the SBRs under scatter correction conditions. Phantom calibration may correct overestimation of the scatter component in the radioactivity of the cerebral cortex as a nonspecific area. However, the calibrated SBRs under scatter correction conditions still showed higher values than those obtained under NOSC conditions. This incomplete calibration may be due to the presence of low-abundance highly-penetrating emissions with a high energy of 529 KeV of ^123^I from distant parts of the body, which would not be encountered in the phantom study. From these phantom calibration findings, the provision of a first-order harmonization correction would resolve the largest part of the differences between different SPECT systems but would not establish full equivalence [9, 36].

There are several limitations to this study. First, the aging effect on subfield measures of striatal SBR has not been investigated. There are conflicting data in the literature on this topic. It has been reported that the DAT decline with aging was more evident at the putamen than at the caudate [24]. Other investigators found that the caudate showed a greater decline than the putamen [25, 27]. The latter may be reasonable because age-related cell loss in the substantia nigra is likely to preferentially affect its dorsomedial part, which projects to the caudate nucleus [29]. However, subfield measures of striatal SBR have not been reported to be superior to whole striatal measures in the differential diagnosis of Parkinsonian syndromes [37]. Second, the lack of an effect of handedness on DAT should be taken with caution because the number of partial or full left-handers was very small and this unbalance with respect to right-handers may have affected the results. An ad hoc investigation with balanced numbers is required to fully elucidate this issue. Third, the age dependency of SBRs was investigated in a cross-sectional study of different individuals. Longitudinal measures in the same individuals may be preferable to obtain age-related SBR changes. Fourth, the number of healthy subjects older than 80 years old was small. Further data collection from healthy subjects in their 80s may be necessary.

## Conclusion

This study provides a large-scale database of [^123^I]FP-CIT SPECT scans from different SPECT scanners in healthy controls across a wide age range and with balanced sex representation. Phantom calibration effectively harmonizes quantitative SPECT data from different SPECT systems under NOSC conditions. Higher DAT availability was found in women than in men. Average age-related declines in DAT availability of 5.3% and 7.5% per decade were found for men and women, respectively. The data collected in this study may serve as a reference database for nuclear medicine centers and for clinical trials using [^123^I]FP-CIT SPECT as an imaging marker.

## Electronic supplementary material

ESM 1 (XLSX 19 kb)
